# Impact of an embedded onco-palliative care clinic on urine drug testing in thoracic oncology

**DOI:** 10.1007/s00520-025-09622-3

**Published:** 2025-06-16

**Authors:** Julia L. Agne, Amanda V. Gusovsky Chevalier, Jason A. Benedict, Nida Khan, Maureen Saphire, Pooja Kumar, Madison M. Grogan, Justin Kullgren, Sachin S. Kale, Jack Stevens, Ann Scheck McAlearney, Carolyn J. Presley

**Affiliations:** 1 Division of Palliative Medicine, The Ohio State University Wexner Medical Center, 1581 Dodd Drive, Columbus, OH 43210 USA; 2 The Center for the Advancement of Team Science, Analytics, and Systems Thinking in Health Services and Implementation Science Research (CATALYST), The Ohio State University College of Medicine, Columbus, OH USA; 3 Center for Biostatistics, The Ohio State University, Columbus, OH USA; 4 Division of Medical Oncology, The Ohio State University Comprehensive Cancer Center, Columbus, OH USA; 5 Department of Pharmacy, The Ohio State University James Cancer Hospital, Columbus, OH USA; 6 Nationwide Children’s Hospital, Columbus, OH USA; 7 Department of Pediatrics, The Ohio State University, Columbus, OH USA; 8 Department of Family and Community Medicine, The Ohio State University, Columbus, OH USA

**Keywords:** Urine drug testing, Cancer, Palliative care

## Abstract

**Purpose:**

Urine drug testing (UDT) is recommended, yet underutilized, for patients receiving opioids for cancer pain. The primary aim of this study was to evaluate the impact of an embedded onco-palliative care clinic on UDT among patients with lung cancer. The number of patients tested, the timing of the first UDT, the incidence of unexplained UDT results, testing frequency, and substances detected on UDT were explored.

**Methods:**

This is a single-institution retrospective study of patients diagnosed with any stage thoracic malignancy who began urine drug testing 1 year before (pre-cohort) and 1 year after (post-cohort) implementation of an embedded thoracic oncology-palliative care clinic in Columbus, Ohio, USA, on September 5, 2018. Confirmatory UDT was routinely ordered for any patient receiving opioids prescribed by palliative care or via ad hoc testing by oncology providers regardless of palliative care referral status.

**Results:**

More patients completed UDT after implementation of an embedded onco-palliative care clinic (pre-cohort, *n* = 61; post-cohort, *n* = 182). Pre-cohort patients began UDT closer to death with median survival of 5.2 months after first UDT (post-cohort, 10.9 months; *p* < 0.0001). While a larger proportion of post-cohort patients completed > 1 UDT (pre, 26.6%, post, 46.7%; *p* < 0.01), there was no significant difference in the proportion of patients experiencing an unexplained UDT result (pre, 9.8% vs. post, 11.0%, *p* = 0.80).

**Conclusion:**

Implementation of an embedded onco-palliative care clinic was associated with a significant increase in use and earlier initiation of UDT among patients receiving care in a thoracic oncology clinic.

## Introduction

Opioids are the primary class of medication used in the treatment of cancer-related pain [1, 2]. As nonprescribed substance use is common among patients with cancer [3], expert opinion and consensus guidelines strongly recommend routine urine drug testing (UDT) for all patients upon initiation and continuation of opioid therapy for cancer-related pain [4, 5]. UDT produces objective data on patients’ use of prescribed controlled medications and nonprescribed substances, which can enhance a provider’s decision-making process by providing information about medication non-adherence and nonprescribed substance use undisclosed by the patient during clinical encounters [6]. Despite consensus guidelines and potential benefits to therapeutic decision-making, UDT throughout cancer care is rare [7]. Moreover, there is no consensus on the appropriate timing and frequency of UDT for patients receiving controlled medications for cancer symptom management [6, 8, 9].

Substance use disorder (SUD) is associated with opioid-related emergency department visits and hospitalizations; therefore, screening for SUD is essential in cancer-related pain management [10, 11]. Survey-based screening tools have been shown to overestimate SUD risk among patients with cancer [12], whereas chromatography-based drug testing, often referred to as confirmatory UDT, is the gold standard for controlled medication monitoring and detection of nonprescribed substance use [13]. Identification of opioid non-adherence or non-medical opioid use (NMOU) is clinically important as opioid-related harms, such as overdose, occur at a higher rate among patients with both solid-tumor and hematologic malignancies [14, 15].

Compared to oncology providers, palliative care specialists are more likely to order UDTs when prescribing opioids for cancer-related pain [7]. Although both specialties routinely prescribe opioids for cancer pain management, differences in opioid-monitoring practices between palliative care and oncology providers have not been elucidated. Through shared clinical workspace, embedded onco-palliative care clinics facilitate closer collaboration between the two specialties as evidenced by increased palliative care referrals and decreased acute healthcare utilization among patients with lung cancer [16, 17]. The effect of embedding palliative care in an oncology clinic on guideline-recommended opioid-monitoring practices, including UDT, has not yet been reported. Therefore, this study aims to examine differences in UDT practices before and after opening an embedded thoracic onco-palliative care clinic for patients with lung cancer.

## Methods

This study was approved by the institutional review board at The Ohio State University Comprehensive Cancer Center in Columbus, Ohio, USA.

### Study cohort selection

**Fig. 1:**
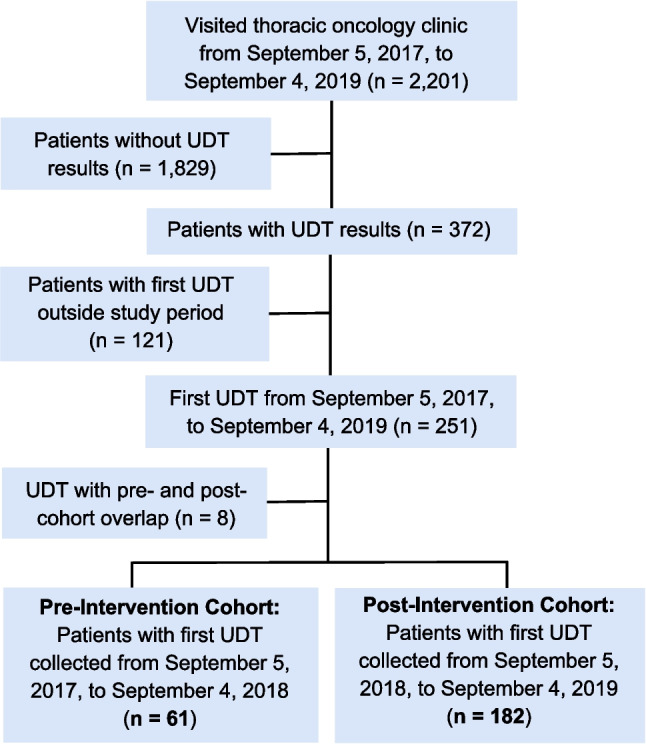
Consort diagram depicting cohort selection for data analysis

This was a single-center retrospective pre-post study that included adult (≥ 18 years old) patients with any stage thoracic malignancy seen in the Thoracic Oncology Clinic and who completed confirmatory UDT either during the year prior to or after the opening of the embedded Thoracic Onco-Palliative Care Clinic on September 5, 2018. Cohort entry (*t* = 0) was defined as the date when a patient began UDT. Patients whose first drug test occurred from September 5, 2017 to September 4, 2018 were assigned to the pre-intervention cohort, while patients who completed an initial UDT from September 5, 2018 to September 4, 2019 were assigned to the post-intervention cohort (Fig. 1). Pre-cohort patients who completed UDT in both standalone and embedded palliative clinic settings were excluded. Each patient was followed for up to 24 months after cohort entry or until death or loss to follow-up, whichever occurred first.

### Palliative clinic structure

Prior to September 2018, all outpatient palliative care referrals at our cancer center were completed at a standalone palliative care clinic located approximately 2 miles from the Thoracic Oncology Clinic. In a standalone clinic, palliative care visits occur in a different location and often on a different day from the patient’s oncology appointments. At the time of this study, the standalone palliative clinic operated 5 days/week with three palliative medicine physicians, four certified nurse practitioners, two palliative clinical pharmacists, one social worker, and full-time nursing and scheduling staff.

On September 5, 2018, we opened an embedded palliative care clinic in thoracic medical oncology to provide coordinated oncology and palliative care for patients with advanced thoracic malignancies [17, 18]. At the time of this study, a single palliative care physician saw patients 2 days/week alongside five thoracic oncologists. In this embedded clinic, palliative care providers share workspace, exam rooms, and clinic resources with oncology clinicians, including a social worker, three nurse case managers, and full-time nursing and scheduling staff.

### Palliative clinic UDT protocol

In the standalone palliative clinic, UDT is completed at every patient’s first palliative clinic visit regardless of risk factors for NMOU or SUD. In the embedded palliative clinic, UDT is completed at a patient’s first palliative clinic visit unless UDT was ordered by the oncology team within the previous 6 months. For opioid management in both clinics, UDT is repeated every 6 months or sooner per provider discretion. During the post-intervention period, oncology providers could order confirmatory UDT, which was encouraged, but not mandatory, for any patient regardless of palliative care referral status.

In this study, UDT consisted of a two-step process in which urine samples were first tested by screening immunoassay followed by confirmatory gas or liquid chromatography-mass spectrometry (GC–MS, LC–MS). As immunoassay UDT is prone to false-negative results with an overall sensitivity of 78.5%, automatic reflex of urine samples to GC–MS/LC–MS increases the sensitivity of UDT results to 84.6% via this two-step process [13]. All urine samples were sent to an external laboratory for the two-step UDT (Dominion Diagnostic Laboratories, LLC; North Kingston, RI). Laboratory and testing costs are covered by Medicare, Medicaid, and in-network private health insurances with no out-of-pocket cost to patients. Samples were tested for amphetamines, anticonvulsants, barbiturates, benzodiazepines, cannabinoids, cocaine, ethanol, and opioids. Testing for additional substances or medications was available upon request.

Patients with UDT results suggestive of SUD were offered referrals to counseling and treatment resources external to the palliative care clinics. Among patients with unexplained UDT results, UDT was repeated at every palliative clinic appointment until hospice referral, death, discontinuation of palliative care, or loss to follow-up. This testing procedure allowed palliative and oncology providers to closely monitor active nonprescribed substance use and medication non-adherence with the goal of providing patients with additional support during cancer treatment.

### Interpretation of UDT results

Palliative-trained clinical pharmacists reviewed all unexpected UDT results, reconciled unexpected results with any facility-administered medications or patient-reported last doses of prescribed medications documented in the electronic medical record (EMR), and coded unexpected UDT results as either explained, unexplained, or inconclusive [19]. Unexpected UDT results were classified as “explained” if a facility-administered or patient-reported medication was not appropriately documented on the testing requisition form. Inconclusive UDT results typically included samples that lacked confirmatory GC–MS/LC–MS testing either due to insufficient sample quantity or failure to request confirmatory testing for substances not included in the standard testing panel. Unexplained UDT included results that could not be reasonably explained by medication reconciliation, prescription drug monitoring program (PDMP) records, or patient-reported medication use documented in the EMR.

In Ohio, marijuana was legalized for medical use in 2016 prior to the start of the pre-intervention cohort [19]. Therefore, cannabinoids and ethanol were coded as explained UDT results, and patients who tested positive for cannabinoids or ethanol were reported as using non-illicit, nonprescribed substances. Palliative clinical pharmacists sub-coded unexplained UDT results as either absence of a prescribed medication, presence of a nonprescribed substance, or a combination of both absence/presence in the same urine sample. Finally, unexplained results were further coded by drug class, including amphetamines, anticonvulsants (gabapentin, pregabalin), benzodiazepines, cocaine, and opioids. Drug classes were not mutually exclusive, as a single urine sample could contain multiple unexplained results (e.g., positive for cocaine and negative for a prescribed opioid). UDT results with no abnormal findings did not require palliative pharmacist review and were coded as “explained” by the research team.

### Data collection and statistical analysis

Patient demographics (age, sex, race/ethnicity, marital status, and county of residence), cancer diagnosis (non-small-cell lung cancer [NSCLC] or small-cell lung cancer [SCLC]), and cancer stage at diagnosis (I–IV) were abstracted from the EMR. Dates of palliative care and oncology appointments, UDT results, last patient contact, and death were also abstracted from the medical record, if applicable. As the Thoracic Oncology Clinic is located in Franklin County, Ohio, patients’ county of residence was further categorized by proximity to the oncology clinic (Franklin County, county adjacent to Franklin County, or county outside both Franklin and the adjacent counties). Palliative care engagement was defined as the number of days between a patient’s first and last palliative clinic visits up to 24 months after cohort entry. Patients who completed only one palliative care appointment were assigned an engagement time of 1 day. Coding of unexpected UDT results was abstracted through manual chart review of UDT interpretation documented by palliative pharmacists in the EMR.

Differences in demographics and UDT variables between the pre- and post-intervention cohorts were assessed using logistic regression for binary variables, either Fisher’s exact test or chi-squared tests for categorical variables, and *T*-test for continuous variables. Survival after initiating UDT (i.e., cohort entry) was calculated for both cohorts and plotted as Kaplan–Meier curves. Log-rank tests were used to assess statistically significant differences in survival curves. Swimmer plots were constructed to descriptively demonstrate longitudinal patterns of unexplained UDT results during opioid management for cancer-related pain. *p*-values < 0.05 were considered statistically significant, and all *p*-values are presented at their nominal levels. Statistical analyses were conducted in SAS 9.4 and R 4.4.

## Results

### Patient characteristics and survival analysis

**Fig. 2:**
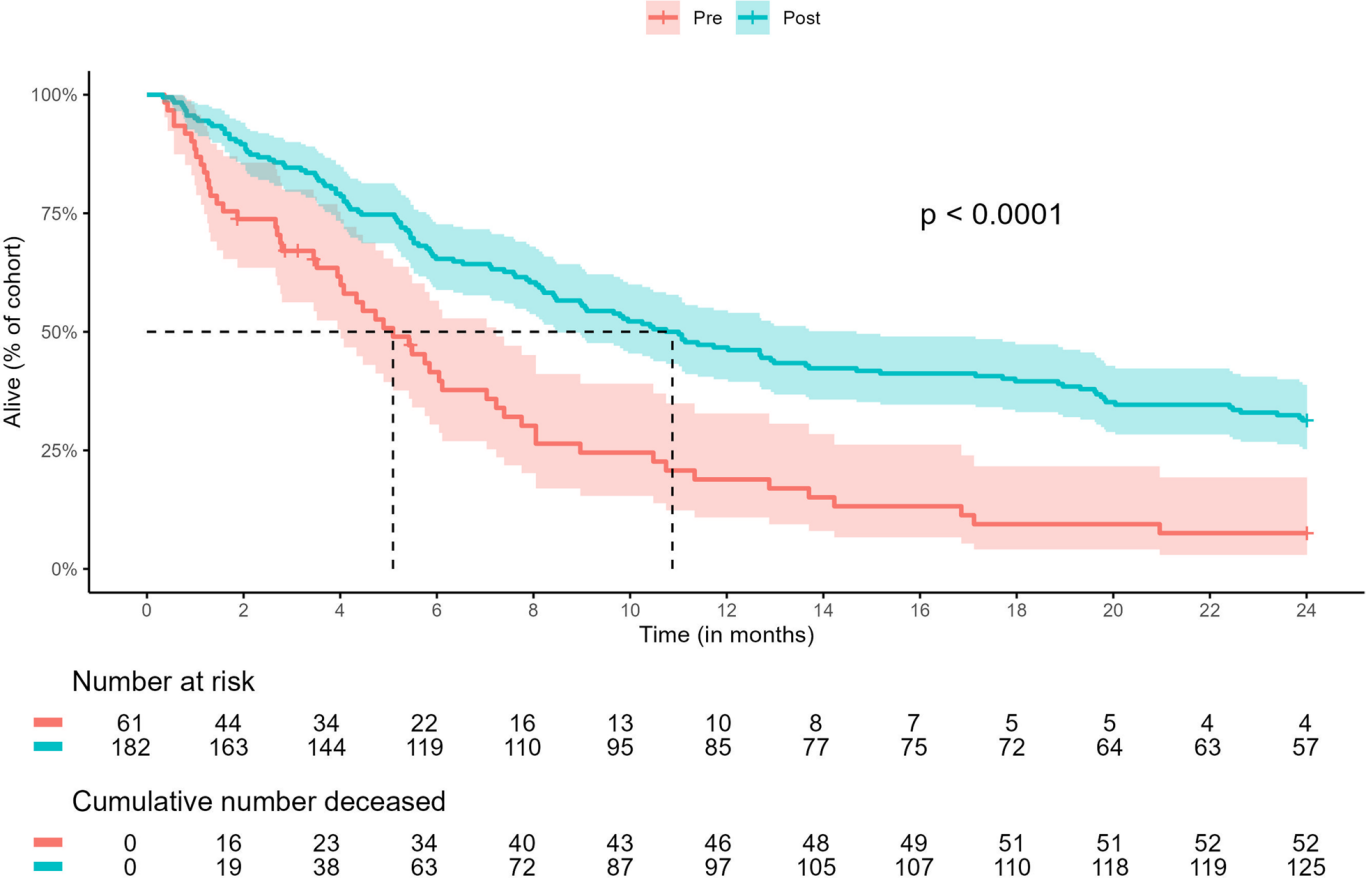
Kaplan–Meier curves depicting survival after first urine drug test by cohort

A total study sample of 243 patients, with 61 patients in the pre-intervention cohort and 182 patients in the post-intervention cohort, were included (Fig. 1). There were no significant differences in age, sex, race/ethnicity, marital status, county of residence, or cancer diagnosis/stage between cohorts (Table 1). Most patients in both cohorts had metastatic disease at cancer diagnosis, defined as either stage IV NSCLC (pre-cohort, 82.0%; post-cohort, 81.8%) or extensive-stage SCLC (pre-cohort, 16.4%; post-cohort, 11.5%). There was a significant difference in mortality, with 85.3% (52/61) of the pre-cohort and 68.7% (125/182) of the post-cohort deceased at 2 years after cohort entry (*p* < 0.01). Median survival after the first drug test was 5.7 months longer in the post-cohort (pre, 5.2 months; post, 10.9 months; *p* < 0.0001), indicating that patients in the pre-cohort initiated UDT closer to death compared to post-cohort patients who began UDT after embedding a palliative provider in the thoracic oncology clinic (Fig. 2).

**Table 1:** Patient characteristics

**Characteristic**^a^	**Pre-intervention cohort** (*n* = 61)	**Post-intervention cohort** (*n* = 182)	***p*-value**^c^
**Age, median (IQR)**	60 (55–67)	61 (55–70)	0.38
**Sex**			0.32
*Female*	34 (55.7)	88 (48.4)	
*Male*	27 (44.3)	94 (51.6)	
**Race/ethnicity**			0.52
*Non-Hispanic White*	50 (82.0)	159 (87.4)	
*Non-Hispanic Black*	9 (14.8)	17 (9.3)	
*Hispanic*	1 (1.6)	4 (2.2)	
*Other*	1 (1.6)	2 (1.1)	
**Marital status**			0.97
*Married*	35 (57.4)	104 (57.1)	
*Unmarried*	26 (42.6)	78 (42.9)	
**Residential location**			0.17
*Within Franklin County*	27 (44.3)	60 (33.0)	
*Surrounding Franklin County*	12 (19.7)	32 (17.6)	
*Outside Franklin and surrounding counties*	22 (36.1)	90 (49.4)	
**Cancer diagnosis/stage**			0.45
*NSCLC stage 1*	3 (4.9)	4 (2.2)	
*NSCLC stage 2*	0 (0.0)	6 (3.3)	
*NSCLC stage 3*	8 (13.1)	21 (11.5)	
*NSCLC stage 4*	40 (65.6)	128 (70.3)	
*SCLC, limited*	0 (0.0)	2 (1.1)	
*SCLC, extensive*	10 (16.4)	21 (11.5)	
**Patient status at cohort end**^b^			< 0.01
*Alive*	4 (6.5)	57 (31.3)	
*Deceased*	52 (85.3)	125 (68.7)	
*Lost to follow-up*	5 (8.2)	–	

*NSCLC* non-small-cell lung cancer, *SCLC* small-cell lung cancer

^a^Presented as *n* (%) unless otherwise indicated

^b^Defined as death status at 2 years after patient’s first UDT (i.e., cohort entry)

^c^Using Fisher’s exact test for categorical variables and Wilcoxon rank-sum test for continuous variables

### Urine drug testing and exposure to outpatient palliative care

There was a significant difference in incidence and timing of UDT related to outpatient palliative care referral (Table 2). Only 24.6% (15/61) of the pre-cohort compared to 39.2% (56/143) of patients in the post-cohort were seen longitudinally by an outpatient palliative care provider for more than 6 months. Among post-cohort patients, 21.4% (39/182) completed UDT ordered by oncology providers without any contact with outpatient palliative care. The proportion of patients completing only one drug test in both cohorts (pre, 73.8% (45/61); post, 53.3% (97/182)) is reflective of the proportion of patients with less than 6 months of palliative care engagement, while the proportion of patients completing > 3 drug tests is significantly different between cohorts (pre, 6.6% (4/61); post, 26.9% (48/182); *p* < 0.01). There was also a significant difference (*p* < 0.01) in the categorical distribution of when a first drug test was completed relative to the date of the palliative care referral order, although the median number of days from palliative referral to UDT was similar between the two cohorts (pre, 17.0; post, 14.0). Among patients receiving outpatient palliative care, 36.3% (52/143) in the post-intervention cohort versus only 14.8% (9/61) in the pre-intervention cohort completed UDT within 1 week of the palliative care referral being ordered.

**Table 2:** Palliative care engagement and urine drug testing results per patient

**Patient characteristics**^**a**^	**Pre-intervention cohort** (*n* = 61)	**Post-intervention cohort** (*n* = 182)	***p*-value**^e^
**Palliative care engagement time**^b^			
*None*	–	39 (21.4)	
*1 day*	13 (21.3)	21 (11.5)	
*> 1 day to 6 months*	33 (54.1)	66 (36.3)	
*6–12 months*	9 (14.8)	31 (17.0)	
>* 12 months*	6 (9.8)	25 (13.7)	
**UDT completion**			< 0.01
*1 UDT*	45 (73.8)	97 (53.3)	
*2 UDT*	12 (19.7)	36 (19.8)	
*3 UDT*	0 (0.0)	18 (9.9)	
*4 UDT*	0 (0.0)	8 (4.4)	
*≥ 5 UDT*	4 (6.6)	23 (12.6)	
**First UDT prior to PC referral**^c^			
*UDT without PC referral*	–	22 (56.4)	
*UDT before PC referral*	–	17 (43.6)	
**First UDT after PC referral**^d^			< 0.01
<* 1 week from PC referral*	9 (14.8)	52 (36.3)	
*1–4 weeks from PC referral*	31 (50.8)	58 (40.6)	
>* 4 weeks from PC referral*	21 (34.4)	33 (23.1)	
**Patients with unexplained UDT results**			
*Any unexplained result during the study*	6 (9.8)	20 (11.0)	0.80
*Occurrence of first unexplained result:*			
*Baseline UDT*	5 (8.2)	10 (5.5)	
*Post-baseline UDT*	1 (1.6)	10 (5.5)	
**Non-illicit, nonprescribed substances**			
*Cannabinoids*	5 (8.2)	19 (10.4)	0.61
*Ethanol*	0 (0.0)	7 (3.9)	0.12

^a^Presented as *n* (%) unless otherwise indicated

^b^Defined as the interval (days) between first and last palliative clinic appointments. Patients with only 1 palliative care appointment were assigned an engagement time of 1 day

^c^Only post-cohort patients (*n* = 39) were able to complete UDT without or prior to a palliative care referral

^d^Only patients with PC referral ordered before first UDT are represented (pre-cohort: *n* = 61; post-cohort: *n* = 143)

^e^Unadjusted *p*-values obtained by logistic regression, *t*-test, or chi-squared test for binary, continuous, and categorical variables, respectively

### Unexpected UDT results by patient

The proportion of patients experiencing an unexplained UDT result was similar in both cohorts (pre-intervention, 9.8% (6/61); post-intervention, 11.0% (20/182); *p* = 0.80; Table 2). As UDT would not alter the incidence of NMOU or SUD, we expected the proportion of unexplained UDT results on patients’ first drug test (i.e., baseline UDT) to be similar between the two cohorts as was observed (pre-post, 5/61 (8.2%) versus 11/181 (6.0%); *p* = 0.56). Sixteen (26.2%) patients in the pre-cohort and 85 (47.0%) in the post-cohort were tested post-baseline. Of the patients with > 1 UDT result, an unexplained result was first detected in 1/16 (6.3%) pre-cohort and 9/85 (10.6%) on post-baseline UDT. As the number of patients completing post-baseline UDT nearly doubled after embedding palliative care in the thoracic oncology clinic, so did the number of unexplained post-baseline results.

**Fig. 3:**
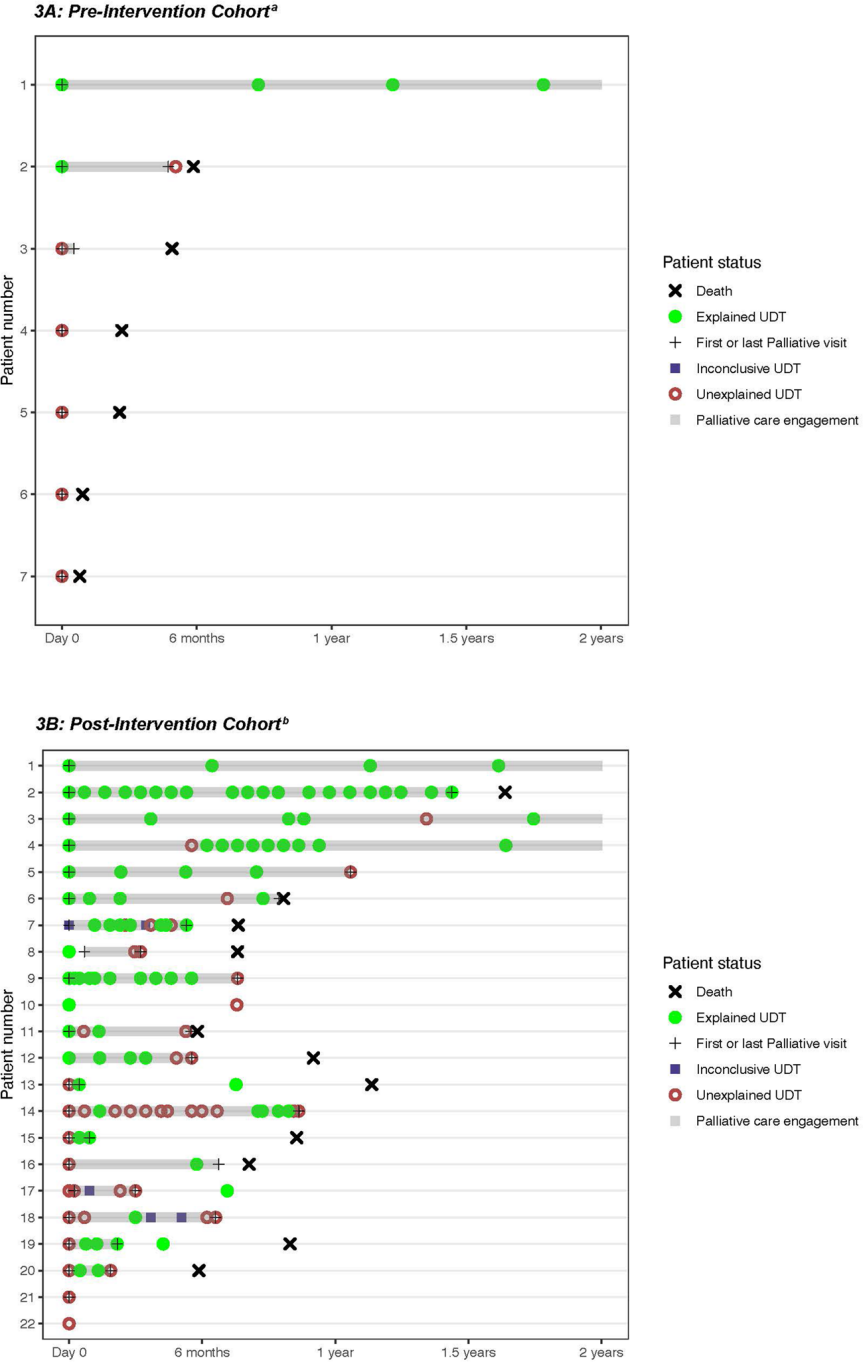
**A, B** Swimmer plots by cohort depicting coded interpretations of urine drug test results, interval time of palliative care engagement, and patient death up to 2 years after the first urine drug test. ^a^*Pre-cohort:* For reference, #1 is representative of a patient with no evidence of unexplained results on routine UDT approximately every 6 months per clinic protocol. ^b^*Post-cohort:* For reference, #1 is representative of a patient with no evidence of unexplained results on routine UDT approximately every 6 months per clinic protocol. For comparison, #2 is representative of a patient with self-reported history of substance use disorder with no evidence of unexplained results on UDT conducted at each clinic visit per clinic protocol

Figure 3 depicts coded UDT results over time for any patient with an unexplained result during the 2-year observation period. Three patients with no unexplained results are included in the swimmer plots for comparison of testing frequency per clinic protocol. The time interval from baseline UDT to first unexplained result ranged from 0 to 154 days in the pre-cohort and 0 to 490 days in the post-cohort. In the pre-cohort (Fig. 3A), all unexplained UDT results occurred within 6 months of patient death. Among patients with unexplained UDT results in the post-cohort (Fig. 3B), 15/20 (75.0%) patients first experienced an unexplained result > 6 months before death; 15/20 (75.0%) also repeated UDT after experiencing an unexplained result.

### Unexplained UDT results by cohort

A total of 49 unexplained UDT results were found in this study (Table 3). There were 6 unexplained UDT results for 6 patients in the pre-cohort and 43 unexplained results for 20 patients in the post-intervention cohort. In both cohorts, similar proportions of unexplained UDT results were due to the absence of a prescribed medication and the presence of a nonprescribed/illicit substance. Opioids were the most common unexplained UDT result (pre, 83.3% (5/6); post, 54.7% (29/46)) followed by benzodiazepines and amphetamines. The presence of cocaine and the absence of prescribed anticonvulsants (gabapentin/pregabalin) were detected as unexplained UDT results only in the post-intervention cohort.

**Table 3:** Summary of urine drug testing results

**UDT result**^**a**^	**Pre-intervention cohort**^f^ (*n* = 93)	**Post-intervention cohort**^f^ (*n* = 419)	***p*-value**^g^
**Coded result by urine sample**			0.39
*Explained*	80 (86.0)	338 (80.7)	
*Unexplained*	6 (6.5)	43 (10.3)	
*Inconclusive*	7 (7.5)	38 (9.0)	
**Urine samples with unexplained results**^b^			0.69
*Absence of prescribed medication*	2 (33.3)	12 (27.9)	
*Presence of nonprescribed substance*^c^	4 (66.7)	26 (60.5)	
*Combination absence/presence*^d^	0 (0.0)	5 (11.6)	
**Unexplained result due to**^e^			
*Amphetamine*	1 (16.7)	5 (9.4)	0.68
Presence	1 (100.0)	5 (100.0)	
*Gabapentin/pregabalin*	0 (0.0)	4 (7.6)	0.45
Absence	0 (0.0)	4 (100.0)	
*Benzodiazepine*	1 (16.7)	11 (20.8)	0.69
Absence	1 (100.0)	5 (45.5)	
Presence	0 (0.0)	6 (54.6)	
*Cocaine*	0 (0.0)	4 (7.6)	0.45
Presence	0 (0.0)	4 (100.0)	
*Opioid*	5 (83.3)	29 (54.7)	0.18
Absence	2 (40.0)	10 (34.5)	
Presence	3 (60.0)	19 (65.5)	

^a^Presented as *n* (%) unless otherwise indicated

^b^Represents 6 unexplained UDT results in the pre-intervention cohort and 45 unexplained UDT results in the post-intervention cohort. Categories do not indicate the number of unexplained results detected in a single urine sample

^c^Includes illicit substances

^d^Represents UDT results with both an unexplained absence of a prescribed medication and presence of a nonprescribed substance detected on a single urine sample

^e^Substances are not mutually exclusive as a single urine sample may have > 1 unexplained result. “Presence” refers to a nonprescribed substance detected in a urine sample. “Absence” refers to non-detection of a substance that was both prescribed and reported by the patient as consumed within the detection window of urine sample collection

^f^Represents the number of tests completed for 61 patients and 182 patients in the pre- and post-intervention cohorts, respectively

^g^Unadjusted *p*-values obtained by chi-squared test

## Discussion

In this study, we implemented confirmatory UDT in a post-intervention group where barriers to palliative care referrals were reduced through an embedded clinic model [17] and oncology providers were empowered to initiate UDT for patients not receiving specialty palliative care. By embedding palliative care, the primary location of UDT shifted from a standalone palliative care clinic to the oncology clinic where patients received cancer treatment. With this shift in testing location, we observed a threefold increase in the number of patients who initiated UDT in the first year of opening an embedded onco-palliative care clinic. While increased use of UDT may be reflective of increased access to outpatient palliative care in the post-intervention cohort [17], this is the first study to demonstrate the impact of an embedded palliative care clinic on the timing of UDT relative to death as a competing event among patients with lung cancer.

Beyond diagnosing SUD, the ability of UDT to detect nonprescribed substances and medication non-adherence may be useful in assessing a patient’s risk for polypharmacy, drug-drug interactions, and underutilization of prescribed medications for symptom management. When UDT is driven by palliative care consultation [7], completion of UDT in a standalone palliative clinic depends on whether a patient is willing or able to receive palliative care in a different location from their primary oncology clinic. Our prior research has demonstrated no effect of clinic model, standalone versus embedded, on when a patient is referred to outpatient palliative care after a lung cancer diagnosis [17]. However, this study shows that pre-cohort patients began UDT at a first palliative care visit in a standalone clinic at a timepoint closer to death compared to post-cohort patients who could complete UDT in the oncology clinic. By waiting for palliative care to initiate UDT in a standalone clinic, there may be delayed recognition of and less opportunity to support patients with active SUD, NMOU, or non-compliance with controlled medications prescribed for symptom management during cancer care.

While there is strong support for drug screening at least once, there is no consensus on repeat testing during opioid therapy for cancer pain management [4, 6, 8, 9]. UDT was repeated more frequently in the post-cohort, with patients completing one additional test, on average, compared to pre-cohort subjects. This is most reflective of the longer survival after the first drug test in the post-intervention cohort, which allows for additional time to repeat UDT during the disease course. As the pre-cohort median survival after the first drug test was less than 6 months, patients receiving palliative care in the standalone clinic were less likely to repeat UDT per clinic protocol. In the post-cohort, where about half of patients completed more than one drug test, half of cases of nonprescribed substance use or controlled medication non-adherence were first detected on a post-baseline UDT result. Pre-cohort patients with unexplained UDT results did not repeat testing and rarely followed up with palliative care. By contrast, post-cohort patients with an unexplained UDT result continued to follow with palliative care alongside their oncology team and often demonstrated relapsing substance use throughout cancer care. Together, these findings demonstrate the importance of repeated testing for patient safety and that a single drug test is insufficient to monitor for nonprescribed substance use or medication non-adherence for patients receiving opioids for cancer-related pain.

We have previously shown that patients are more likely to be referred for and complete a palliative care consultation when a palliative provider is embedded in an oncology clinic [17]. However, increased UDT in the post-cohort is only partially explained by increased access to palliative care, as more than one-fifth of post-cohort subjects completed UDT without a palliative care consultation. As the proportion of oncologist-initiated UDT is notably higher in this study than previously described [7], we hypothesize that the presence of an embedded palliative care physician may have nudged [20] oncology providers to utilize UDT through case review, direct communication of unexplained results, and UDT interpretation by palliative clinical pharmacists. In the future, two nudge interventions that hold promise to further expand the use of UDT include (a) order sets [20], in which the electronic medical record system automatically selects this test whenever an opioid is prescribed while allowing the provider to decline that recommendation, and (b) peer comparisons [21], in which providers are given periodic feedback regarding how frequently they order this test relative to similar clinicians’ decisions. Engagement of oncology clinics in drug screening practices is crucial, as patients with nonprescribed substance use or medication non-adherence may be missed by screening protocols that are only employed by palliative care providers.

There were differences, albeit not statistically significant, in types of unexplained UDT results detected between the two cohorts. Opioids—both the absence of prescribed (non-adherence) and presence of nonprescribed opioids—contributed most to the unexplained results in both cohorts. Compared to the pre-cohort, unexplained UDT results were more heterogeneous among post-cohort patients. Similar proportions of patients in both cohorts tested positive for cannabinoids, although delineation of whether cannabinoids were obtained through legal dispensaries versus illegal sources was beyond the scope of this study. Alcohol use was detected on UDT only in the post-cohort due to a difference in ethanol testing between the two cohorts. Ethanol metabolites (ethyl glucuronide, ethyl sulfate) were added to the testing panel during the post-intervention period, which detects alcohol consumption up to 80 h (3.33 days) prior to urine collection. Although patients are counseled to avoid concomitant use of opioids and alcohol, the clinical significance of modest alcohol consumption with chronic opioid therapy is not well understood.

As this study was conducted at a large academic medical center with robust outpatient palliative care resources, we recognize several limitations that may limit the generalizability of these findings. First, the sample size was small, particularly in the pre-intervention cohort, which decreased the power of the study to detect differences in UDT results between cohorts. Second, we did not calculate the overall proportions of thoracic oncology patients completing UDT (i.e., screening rate) because many patients were followed in the Thoracic Oncology Clinic during both the pre- and post-intervention time periods. To minimize the effect of pre-post cohort overlap, we defined the cohorts based on when a patient’s first drug test occurred to describe the effect of an embedded onco-palliative care clinic on UDT utilization. As data was only collected on patients completing UDT, this study does not provide any insight on patients who were prescribed opioids without guideline-concordant UDT. While the median time intervals from palliative care referral to first UDT were similar between the cohorts, the survival analysis supports our conclusion that the post-cohort began UDT at an earlier timepoint relative to death. Finally, patient outcomes, including medication changes, after an unexplained UDT result were beyond the scope of this study and are an area of future research. The economic impact and patient acceptance of recurrent UDT are also important factors that need to be considered in the implementation of drug screening during cancer care, which were not explored in this study.

Palliative care is leading the effort in caring for patients with cancer and substance use disorder [22–25]. Embedding palliative care in a thoracic oncology clinic facilitated the expansion of UDT for patients with lung cancer in concordance with national consensus guidelines [4] and is a viable strategy to improve patient safety in opioid cancer pain management. Providers must consider the resources needed to support patients who demonstrate nonprescribed substance use or medication non-adherence detected via confirmatory UDT. This study highlights how UDT, implemented in a thoracic medical oncology clinic, can identify nonprescribed substance use or medication non-adherence earlier in a patient’s disease course compared to UDT in a standalone palliative care clinic. Early detection is the first step in helping clinicians to better support patients facing SUD, NMOU, or medication non-adherence during cancer care.

## Data Availability

De-identified data is available upon request through a data use and sharing agreement with The Ohio State University Wexner Medical Center.
